# Come together: case specific cross-institutional cooperation of youth welfare services and child and adolescent psychiatry

**DOI:** 10.1186/s13034-019-0294-z

**Published:** 2019-08-29

**Authors:** Judith Mack, Sina Wanderer, Michael Kölch, Veit Roessner

**Affiliations:** 1 Department of Child and Adolescent Psychiatry, Faculty of Medicine Carl Gustav Carus, TU Dresden, Fetscherstraße 74, 01307 Dresden, Germany; 2 Department of Child and Adolescent Psychiatry, Neurology, Psychosomatics and Psychotherapy Rostock University Medical Center, University of Rostock, Rostock, Germany

**Keywords:** Cross-institutional cooperation, Youth welfare services, Child and adolescent psychiatry, Mental health, Health care, Social services

## Abstract

**Background:**

Due to the increasing rate of children and families who require support from both youth welfare services and from mental health services, a solid cross-institutional cooperation is needed to provide coordinated and integrated help. Studies involving not only qualitative, but also quantitative information from both services regarding not only general, but also case specific views on cross-institutional cooperation and psychosocial needs are lacking.

**Methods:**

Hence, we collected data from *n* = 96 children and families who received support from youth welfare office (YWO) and child and adolescents psychiatry (CAP) simultaneously. In a longitudinal survey, we assessed the evaluation of case specific cross-institutional cooperation and psychosocial needs by employees of YWO and CAP as well as descriptive data (including psychopathology of children) over a 6-month period. Repeated-measures ANOVAs were conducted to assess the effects of time and institution (YWO/CAP) on employees’ evaluation of case specific cross-institutional cooperation and psychosocial needs as well as children’s psychopathology.

**Results:**

The data showed that generally YWO employees rated the case specific communication better than CAP employees. Furthermore, CAP employees estimated psychosocial needs higher than YWO employees did. The employees’ evaluation of total case specific cross-institutional cooperation did not differ between the employees of both institutions; it further did not change over time. The case specific evaluations did not correlate between the case responsible employees of YWO and CAP.

**Conclusion:**

The data showed satisfaction with the case specific cross-institutional cooperation in general, but meaningful differences in case specific ratings between both institutions indicate the possibility and need for improvement in daily work and cooperation as well as in regulations and contractual agreements. The implementation of more exchange of higher quality and transparency will ensure smoother cross-institutional cooperation. Future research should pursue this topic to convey the need for further improvement in cross-institutional cooperation into decision-making processes and to evaluate the success of innovative projects in this field.

## Introduction

Worldwide up to every fifth child^1^ is at risk to become mentally ill [1–4]. Risk factors for the development of psychiatric disorders, such as low socioeconomic status, parental mental health disorders, single-parenting or out of home-living, have been identified and are widely discussed [5, 6]. Often, children in psychiatric treatment and their families receive support from youth welfare services (e.g. family assistance, residential care) [7]. Vice versa, a high number of children and families receiving support from youth welfare services need or receive support also from mental health services, particularly child and adolescent psychiatry (CAP) [8–11]. Yet, support from youth welfare services may be complicated by mental health problems, e.g., it is indicated that especially externalizing problems are predictive for placement breakdown in foster care [12] and can massively stress the social work in residential care [13]. Considering those facts, it is evident that many families need and receive support from both youth welfare services and CAP, often simultaneously. Due to this common involvement, the necessity arose that support provided by the different systems is planned and coordinated cooperatively, to promote child development and to avoid support discontinuation.

Over the past years, national and international literature reviews and guidelines highlighted the needs, difficulties, improvements and chances in cross-institutional cooperation^2^ particularly in the context of child and family support [14–17]. Additionally, previous, mostly qualitative studies conducted expert interviews on cooperation [18–22] and consistently reported that clear aims, mutual respect, common language and definitions, permission to collaborate, and time for communication and information sharing are important factors for successful cooperation [15, 18, 19]. Despite the knowledge of these factors, there are ongoing difficulties to transfer them into daily work, collaboration and structures [21].

There is a paucity of studies that not only qualitatively but also quantitatively evaluated cooperation at the intersection of collaborating help institutions, including youth welfare services, CAP, services for child protection etc. [22–24]. In the few existing studies supportive factors for good cooperation were indicated: written agreement of cooperation, case managers to coordinate cases independent of institutions and mutual knowledge transfer [22, 23]. Within the field of residential youth welfare services, in the study of Müller-Luzi and Schmid [25], employees of residential care on the one hand stated that the cooperation with the CAP was usually satisfying. On the other hand, employees of residential care expressed the need for better exchange, information flows and mutual esteem. Corresponding interviews with employees of CAP as well as descriptive and quantitative information from families were not reported [25]. Additionally, only few studies exist that included descriptive as well as quantitative information from both children and families and the institutional employees. Moreover, most existing studies only focus on residential youth welfare services, reporting a lack of studies looking on the broader range of support by the youth welfare system. One study observed the development of children’s mental health longitudinally based on the intensity of cooperation (such as cross-training of staff, working with youth welfare office (YWO), development of agreement) [26]. The authors found that greater intensity of cooperation was associated with improvements in children’s mental health measured by the Child Behavior Checklist (CBCL), within a 36 months period. Darlington et al. [18, 27] surveyed employees from child protection and (child and adult) mental health services in regard to cooperation in *n* = 300 cases, using self-designed questionnaires. They found that in about half of the cases employees reported positive experiences with cooperation. Hence, difficulties in cooperation were stated in 50% of cases, such as non-shared information, confusion in role clarity/case leadership, different/conflicting goals, and unrealistic expectations. For 12% of the study cases employees reported improvements in child treatment due to good cooperative exchange of information. Unfortunately, the concerned children and families took not part in the study and cooperating employees of both institutions were not surveyed in a case specific manner, i.e. reports from both institutions on the same case were not linked with each other. But this is important, because the quality of cooperation in each single case contributes to the overall attitude towards the cooperating institution and vice versa. Furthermore, to assess and detect differences in cross-institutional evaluation of the case specific cooperation may foster stronger future cooperation.

In Germany, the YWO, as part of the youth welfare services, is a local agency with the duty to protect the welfare of children and organize help services for children and families, such as consultation, family assistance, day groups and residential living. The structure and responsibilities of the YWO are regulated nationwide by the Children and Youth Welfare Act (German Social Code—Book VIII, for further information see [28]). The CAP Dresden comprises outpatient, day patient and inpatient clinics with different treatments, such as consultation, psychotherapy (individual, group setting), day patient or inpatient treatment and medication, depending on the psychiatric disorder, severity, social functioning, etc. Although both, YWO and CAP frequently have common patients and see the necessity for cooperation and meetings (e.g. to coordinate and adjust support measures or ways of information), there exists no contractual agreement at federal level how to organize this cooperation. In addition, restricted financial and temporal resources in both systems limit such cooperation plans.

Even though the literature discusses supportive factors for improving cooperation, barriers and problems still exist in cooperation that hinder the optimal or at least healthy development of the concerned child.

Therefore, the aim of the present study was (1) to assess employees’ evaluations of case specific cooperation in the common support of children that receive any kind of support from YWO during treatment in the CAP and (2) of their psychosocial needs. The assessment of the psychopathology of the children to describe this special group also established the possibility to (3) examine possible links between evaluations of by employees of YWO and CAP and psychopathology of the children.

## Method

### Design

This study was part of the project *Evaluation of the Agreement of Cooperation between Youth Welfare Office and Child and Adolescent Psychiatry in Dresden*, and approved by the ethics committee of the Technische Universität Dresden, Germany. To improve cooperative processes, in the year 2013 YWO and CAP Dresden implemented an *Agreement of Cooperation,* which was partly monitored and evaluated. A detailed description of the *Agreement of Cooperation* and the aforementioned evaluation project has already been published elsewhere [29].

For the present project, children and parents who received any kind of support from YWO during inpatient, day patient or outpatient treatment in the CAP were surveyed as well as their case responsible employees from YWO and CAP. The survey was of longitudinal design with three assessment time points (T1–T3). The intervals between these evaluations were 3 months on average.

### Participants

Families of subsample 1 were recruited via phone from a list of current patients of the CAP. Children and their parents gave written informed consent for participation and access to medical reports including permission for investigators to contact the case responsible YWO and CAP employees. Thereafter, the corresponding case responsible employees of YWO and CAP were contacted via phone or (e-)mail. For participation, families received a small expense allowance for each assessment.

**Fig. 1:**
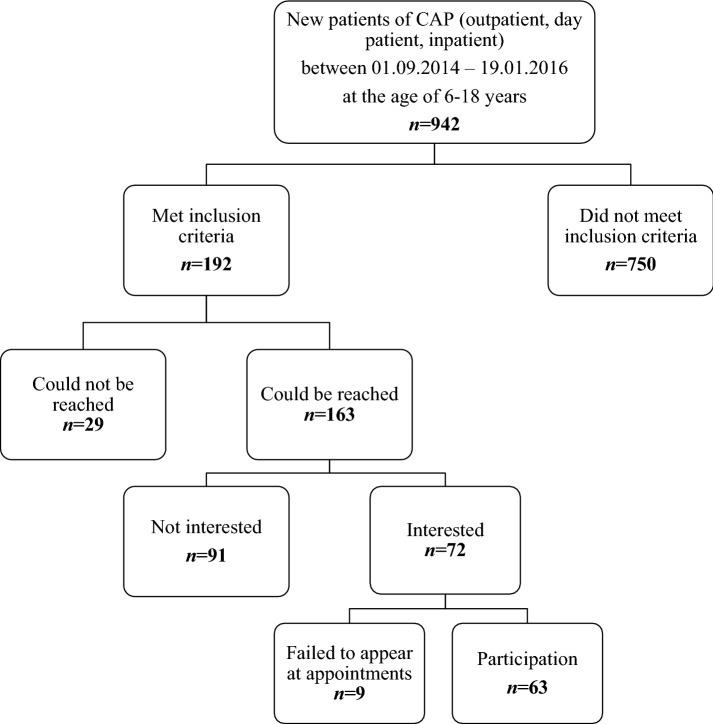
Recruitment process. CAP = Department of Child and Adolescent Psychiatry of the Technische Universität Dresden

About 20% of newly administered patients at the CAP (between September 2014 and January 2016; outpatient, day patient or inpatient) met the inclusion criteria, of which 38% (*n* = 72) were interested. Nine of them did not appear at the first appointment and could not be reached anymore. Finally, *n* = 63 (subsample 1; 33% of the patients who met the inclusion criteria) participated in our survey (Fig. 1).

**Fig. 2:**
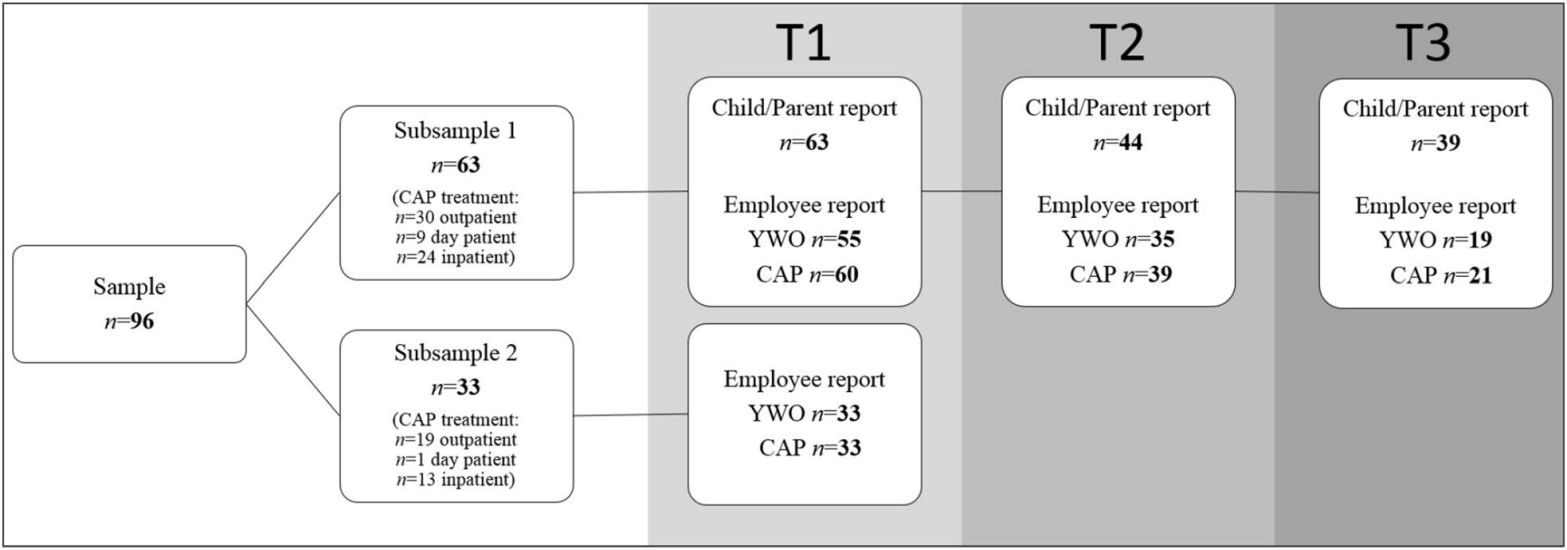
Sample composition and case numbers throughout the study. Subsample1 = children and parents as well as the case responsible employees of YWO and CAP were questioned. Subsample2 = only employees were questioned. CAP treatment setting at T1. YWO = Youth welfare office. CAP = Child and adolescent psychiatry. T1–T3 = times of measurement with approximately 3 months intervals

We additionally included a subsample 2 (Fig. 2), including *n* = 33 cases without direct survey of the families, but whose case responsible employees of YWO and CAP reported about the case specific cooperation, and whose medical reports were surveyed anonymously. In accordance to §34 Abs. 1, Sächsisches Krankenhausgesetz (hospital law, Saxony), no written consent was necessary for subsample 2. Both subsamples showed no differences in age, intelligence (IQ) and psychopathology (all *p* > .066). Figure 2 presents the case numbers over the three time points of measurement (T1, T2, and T3), illustrating the sample size variation over time due to missing data and dropouts (cf. 2.4 Data Analysis).

Altogether, we included data of *n* = 96 cases in the study (43% female, 57% male; *n* = 63 subsample 1, *n* = 33 subsample 2) who received support from YWO and CAP simultaneously. The mean age of participants was *M* = 12.97 years (*SD* = ± 3.17; 28% < 11 years). At T1, 51% of these *n* = 96 cases received outpatient, 10% day patient, and 39% inpatient treatment of CAP (Fig. 2). While *n* = 50 parents in subsample 1 agreed to complete questionnaires, *n* = 13 parents only agreed that we could consult the medical reports.

Twenty-four cases dropped out between T1 and T3, *n* = 16 because they did no longer receive services from CAP (Fig. 2), *n* = 8 cases for undisclosed reason. The dropped out children did not differ from those who continued to participate until T3 in age, IQ, CBCL and YSR *T*-score (all *p* > .227).

All case responsible employees of YWO and CAP were contacted to provide feedback to the investigators. For the *n* = 96 cases, we received at T1 *n* = 88 reports of case responsible employees from YWO and *n* = 93 from CAP (Fig. 2). The employees were not paid for participation. Due to overlapping responsibilities of employees for several cases at one time, some employees evaluated more than one case.

### Measures

#### Assessment of descriptives

Parents provided information on sociodemographic data, child’s place of residence, parents’ relationship status, history of mental disorders in the family (siblings, parents, and grandparents), and their impression on the case specific cooperation of both institutions. Together with available medical reports, we complemented the parents’ information and assessed the child’s psychiatric diagnosis, IQ, and CAP treatment setting (inpatient, day patient or outpatient). In the CAP, all children passed an extensive diagnostic procedure including a physical examination, a comprehensive anamnesis, several clinical diagnostic assessments (e.g. semi-standardized interviews and clinical questionnaires), and, if reasonable, neuropsychological tasks and behavioral observation at home and in school. Finally, ICD-10 [30] diagnoses were established based on the consensus of a multi-professional team directed by a board-certified child and adolescent psychiatrist.

Furthermore, we asked families to fill out some questionnaires that are part of an existing test battery for quality assurance in residential care. Two of them are the parent rating form *Child Behavior Checklist* (CBCL) [31] and the corresponding *Youth Self Report* (YSR) [32] that assessed the general psychopathology of the children, in addition to the child’s psychiatric diagnosis.

The CBCL [31] and YSR [32] are widely used and established measures for the assessment of behavioral and emotional problems of children (aged 4–18 years). Both, parents and children above the age of 11 years were asked to rate behavioral and emotional problems of the last 3 months on a 3-point Likert scale (0 = not true, 1 = somewhat or sometimes true, 2 = very true or often true). In the following analyses, the global scales of the CBCL and the YSR (*total problems score*, *externalizing*, and *internalizing problems*) were used. For these global scales, *T*-scores of 64 or higher are considered clinically relevant [33–36].

#### Evaluation of cooperation

To evaluate the cooperation between YWO and CAP there is no established instrument as well as none with studies on its psychometric properties. Therefore, we used previous descriptions of process and outcome variables in studies using instruments of cooperation and quality management within the youth welfare and healthcare sector cooperation (acc. [37–39]) to develop the instrument, which consisted of six topics (personal information, information about the case, and different sections in light of cooperation: professional attitude, case specific communication, case specific process, satisfaction with aspects of case specific cooperation). Experienced case responsible employees of YWO and CAP had been involved in the development process. Most items were closed questions with a 6-point Likert-Scale (e.g. *How well are agreements on responsibilities and work assignments of the professional employees regulated?* 1 = *very poor* to 6 = *excellent*).

For the evaluation of the case specific cooperation between YWO and CAP, two scores based on some items of abovementioned topics were created, i.e. items out of the topics case specific communication and of satisfaction with aspects of case specific cooperation (Table 1).

**Table 1:** Items of the self-developed questionnaire for YWO and CAP employees used in the present study

Score	Items	Rating
Case specific communication	How successful was the development of a common problem comprehension among the professional employees (themes/issue domains)?	1 = very poor to 6 = excellent
	How transparent have the tasks and capacities of the professional employees been made in the context of case specific cooperation?	1 = very poor to 6 = excellent
	How well are agreements on responsibilities and work assignments of the professional employees regulated?	1 = very poor to 6 = excellent
	How would you estimate the mutual exchange between professional employees regarding successful aspects and errors of the cooperative process?	1 = very poor to 6 = excellent
	How understandable and comprehensible would you estimate the view of the case responsible employee of YWO/CAP)	1 = very poor to 6 = excellent
Total case specific cooperation	Overall, how well did the planning and arrangements of specific help succeed?	1 = very poor to 6 = excellent
	Overall, how well did the communication between the professional employees succeed?	1 = very poor to 6 = excellent
	Overall, how successful was the specific cooperation between the professional employees?	1 = very poor to 6 = excellent
Psychosocial needs	How would you estimate the child’s psychosocial needs?	1 = extremely low to 6 = extremely high

The scores were built on basis of the average of the corresponding items

To assess the perspectives of the YWO and of CAP regarding the *psychosocial needs* of the common case, the employees rated the item *How would you estimate the child’s psychosocial needs?* on a scale from 1 = extremely low to 6 = extremely high.

The score *case specific communication* presents the mean of five items about several aspects of communication (Table 1). The score *total case specific cooperation* presents the mean of three items on how well the case specific cooperation worked in general (Table 1). Both scores vary between 1 = *very poor* and 6 = *excellent* (Table 1). We calculated Cronbach’s alpha of the score *case specific communication* and *total case specific cooperation* for our sample of YWO employees and CAP employees, respectively. The internal consistency of *case specific communication* was .81 for YWO employees and .85 for CAP. The internal consistency of *total case specific cooperation* was .63 for YWO employees and .75 for CAP.

### Data analysis

As mentioned above, in the acquired data values are missing due to dropouts and unanswered items. Thus, systematical errors could occur in statistical inference if data are not Missing Completely at Random (MCAR) [40]. In this analysis the assumption of Missing at Random (MAR) was made, saying the probability to be missing does not depend on the unobserved data [40, 41]. Based on this understanding, population values can be calculated with adequate auxiliary variables that correlate highly with the outcome variable (cf. [42]). We imputed values using regression imputation with a normally distributed residual term (cf. [43]). Correlating variables of *τ* ≥ 0.3 (Kendall’s tau), including time of measurement, age at T1, and gender were used. Since there is more than one time of measurement we used the PAN-algorithm [44].

After imputation the data set included *n* = 96 data points of the CBCL *total problems score* and of the employees’ case specific evaluations (*n*_*YWO*_ = 96; *n*_*CAP*_ = 96) as well as *n* = 69 data points of the YSR *total problems score (n* = 27 of the children were younger than 11 years and did not answer the YSR). We did not impute any family characteristics or additional information from parents or medical reports. Therefore, sample sizes varied dependent on which variable was considered.

Besides descriptive analyses for each variable of interest, we calculated repeated-measures ANOVAs with time of measurement (T1–T3) and institution (YWO vs. CAP) as within-subjects factors for each of the dependent variables *psychosocial needs*, *case specific communication,* and *total case specific cooperation*. Effect sizes are given with partial eta-squared. To identify specific relations and differences between various variables, Pearson correlation coefficients, correlation for paired samples, and *t*-tests (for paired or independent samples) were computed.

All data analyses were conducted with IBM SPSS statistics, version 24. Test requirements were checked and confirmed, and calculations were based on a significance level of 5%.

## Results

### Descriptives

#### Family characteristics

**Fig. 3:**
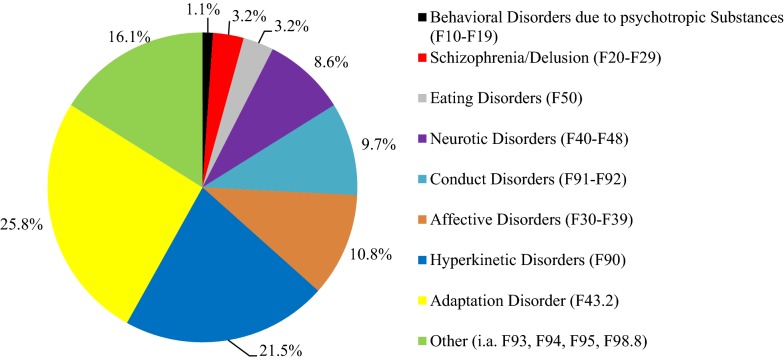
Percentage distribution of initial psychiatric diagnoses (with ICD-10 codes) of the participating children (*n* = 93)

The characteristics of children and their families, who received any kind of support from YWO and CAP simultaneously, were as follows at T1: Eighty-three percent of *n* = 86 parents were separated and 7% had never lived together. Sixty-two percent (n = 59) of the *n* = 96 children stayed with their biological single parent (54% with their biological mother, 8% with their biological father), 15% with both biological parents, 11% with one biological parent and a stepparent, 7% lived in residential care and 5% with grandparents, adoptive or foster parents. The children’s mean IQ was 97 (*n* = 73; *SD* = ± 14.18). All cases (*n* = 96) had an initial psychiatric diagnosis (a prerequisite to receive services from CAP). Figure 3 shows the percentage distribution of *n* = 93 cases; specific diagnoses of *n* = 3 cannot be presented due to missing data in medical reports. Available data of *n* = 79 cases showed that 79% had one to three abnormal psychosocial situations recorded with the Axis V of the ICD-10, 6% had none. The three most psychosocial abnormalities were abnormal environment (60%), mental disorder, deviance or handicap in child’s primary support group (26%), and inadequate or distorted familial communication (18%).

At T1, 51% of the *n* = 96 cases received outpatient treatment and 49% inpatient or day patient treatment of the CAP. Available information of *n* = 80 of these cases showed that 90% (*n* = 72) have been treated in both, psychiatric outpatient as well as inpatient/day-patient settings. Looking at the kind of support from the YWO at T1, 41% of the *n* = 96 cases received support at their family home (e.g. family assistance, social worker for teenage child), 14% got support in the afternoon (e.g. day groups) or received residential care, 10% received other forms of support (combinations of services), and 24% were in the initiation phase for getting support. For 12% we have no exact data.

There was a high rate of mental disorders in the families assessed in this study. Fifty-five percent of mothers, 31% of fathers, 38% of siblings and 24% of grandparents were previously diagnosed with a mental disorder. The parents’ contentment with the cooperation of YWO and CAP was at T1 at *M* = 4.59 (*n* = 49; *SD* = ± 1.14; range 1 = *very poor* to 6 = *excellent*). During the time of receiving support from both institutions, 25% of 47 families never had an appointment with YWO and CAP simultaneously (e.g. to coordinate and adjust the different support measures).

#### Psychopathology

The mean CBCL *total problems score* was *M* = 67.05 (*n* = 96; *SD* = ± 11.68) at T1 and decreased over time (*F*(2, 190) = 15.65; *p* < .001; partial *η*^*2*^ = .141; Table 2). The post-hoc *t*-tests revealed differences between each time of measurement (T1 vs T2: *t*(95) = 2.52, *p* = .013; T2 vs. T3: *t*(95) = 3.08, *p* = .003; T1 vs. T3: *t*(95) = 5.51, *p* < .001). Table 2 shows that the scores of CBCL *externalizing* and *internalizing problems* scales both decreased. Post-hoc tests for *externalizing problems* scores revealed decrease between all time points of measurement (T1–T2: *t*(95) = 2.56; *p* = .012; T2–T3: *t*(95) = 3.41; *p* = .001; T1–T3: *t*(95) = 3.90; *p* < .001). For the *internalizing problems* scores, the post-hoc tests showed decrease between T1 and T3 (*t*(95) = 5.58; *p* < .001) as well as T2 and T3 (*t*(95) = 3.82; *p* < .001). The decrease between T1 and T2 was only trending towards statistical significance (*p* = .059).

**Table 2:** Global scales scores of CBCL and YSR over time

Global scales	*n*	T1	T2	T3	*F*	*η* ^*2*^
		*M* (± *SD*)	*M* (± *SD*)	*M* (± *SD*)		
Total problems score						
CBCL	96	67.05 (11.68)	65.14 (11.44)	62.59 (12.0)	15.65***	.141
YSR	69	61.17 (9.60)	59.62 (9.91)	57.07 (10.40)	13.49***	.166
Externalizing problems						
CBCL	96	65.11 (14.30)	63.67 (13.04)	61.55 (12.25)	12.518***	.116
YSR	69	56.87 (10.54)	53.99 (9.74)	52.83 (9.63)	18.416***	.213
Internalizing problems						
CBCL	96	65.74 (9.66)	64.10 (9.87)	61.18 (9.57)	16.103***	.145
YSR	69	61.48 (11.62)	58.68 (11.22)	60.25 (12.05)	3.973*	.055

T1–T3 = time of measurement; M = mean; SD = standard deviation; CBCL = child behavior checklist; YSR = youth self report; *η*^*2*^ = partial Eta-squared. *p < . 05; ***p < .001

The mean of the YSR *total problems scale* was *M* = 61.17 (*n* = 69; *SD* = ± 9.60) at T1 and decreased as well (*F*(2, 136) = 13.49; *p* < .001; partial *η*^*2*^ = .166; Table 2). Post-hoc *t*-tests indicated decrease between T2 and T3 (*t*(68) = 3.42; *p* = .001) as well as between T1 and T3 *(t*(68) = 4.97; *p* < .001). The decrease between T1 and T2 was only trending towards statistical significance (*p* = .062). Table 2 shows that the scores of YSR *externalizing* and *internalizing problems* scales both decreased. Post-hoc tests for *externalizing problems* scores revealed decrease between T1 and T2 (*t*(68) = 4.26; *p* < .001) as well as T1 and T3 (*t*(68) = 5.54; *p* < .001). For the *internalizing problems* scores, the post-hoc tests showed decrease between T1 and T2 (*t*(68) = 2.82; *p* = .006).

### Evaluation of case specific communication and total case specific cooperation

At T1, the evaluation of the *case specific communication* was *M* = 4.60 (*SD* = ± .88) rated by employees of YWO and *M* = 4.33 (*SD* = ± .85) by employees of CAP (Table 3; 1 = *very poor* to 6 = *excellent*). The repeated-measures ANOVA with institution (YWO vs. CAP) and time of measurement (T1–T3) as within-subjects factors showed a main effect of institution (*F*(1, 95) = 10.06; *p* = .002; partial *η*^*2*^ = .096), but no main effect of time of measurement nor an interaction effect (both *p* > .333). Hence, ratings did not change over time and the YWO rated the case specific communication better than CAP employees did (Table 3). The evaluation of the *case specific communication* by YWO and CAP employees did not correlate with each other at any time (for paired samples: all *r* < .162; all *p* > .114).

**Table 3:** Evaluations by YWO and CAP

	Psychosocial need		Case specific communication		Total case specific cooperation	
	YWO	CAP	YWO	CAP	YWO	CAP
	*M* (± *SD*)	*M* (± *SD*)	*M* (± *SD*)	*M* (± *SD*)	*M* (± *SD*)	*M* (± *SD*)
T1	4.43 (1.13)	4.77 (.76)	4.60 (.88)	4.33 (.85)	4.56 (.89)	4.41 (.87)
T2	4.29 (1.26)	4.77 (1.00)	4.62 (.88)	4.24 (.85)	4.51 (1.03)	4.32 (.86)
T3	4.15 (1.29)	4.63 (1.15)	4.63 (.81)	4.24 (.93)	4.48 (.89)	4.28 (.89)

rmANOVA	F	*η* ^*2*^	F	*η* ^*2*^	F	*η* ^*2*^
Group	12.13**	.113	10.06**	.096	2.37	.024
Time	5.79**	.057	.312	.003	1.99	0.021
Group*Time	.693	.007	1.10	.011	.115	.001

T1–T3 = time of measurement; M = mean; SD = standard deviation; YWO = youth welfare office; CAP = child and adolescent psychiatry. Interpretation of values: psychosocial need =  1 = extremely low to 6 = extremely high; case specific communication and cooperation = 1 = very poor to 6 = excellent. NYWO = 96. NCAP = 96. η^2^ = partial Eta-squared. **p < .01

The *total case specific cooperation* at T1 was rated with a mean of *M* = 4.56 (*n* = 96; *SD* = ± .89; range 1 = *very poor* to 6 = *excellent*) by YWO employees and with a mean of *M* = 4.41 (*n* = 96; *SD* = ± .87) by CAP employees (Table 3). The repeated-measures ANOVA with institution (YWO vs. CAP) and time of measurement (T1–T3) as within-subjects factors revealed neither main effects on the estimation of *total case specific cooperation* nor an interaction effect (all *p* > .127). Thus, the scores maintained stable over time and there were no rating differences between YWO and CAP. The evaluation of the *total case specific cooperation* did not correlate between the case responsible employees of YWO and CAP at any time (correlation for paired samples: all |*r*| < .055; all *p* > .595).

Additionally, neither the scores of *case specific communication* nor those of the *total case specific cooperation* of both institutions correlated with the CBCL or YSR scores or their development over time.

### Evaluation of psychosocial needs

Case responsible YWO employees estimated the children’s *psychosocial needs* with a mean of *M* = 4.4 (*n* = 96; *SD* = ± 1.13; range 1 = *extremely low* to 6 = *extremely high;* Table 3) at T1, CAP employees rated with a mean of *M* = 4.8 (*n* = 96; *SD* = ± .76). There was no correlation between the scores of *psychosocial needs* given by YWO as well as CAP employees with *T*-scores of the CBCL (all |*r*| < .175; all *p* > .088) and the YSR (|*r*| < .191; all *p* > .116) at T1.

The repeated-measures ANOVA with institution (YWO vs. CAP) and time of measurement (T1–T3) as within-subjects factors revealed main effects of both factors on the estimated *psychosocial needs* (institution: *F*(1, 95) = 12.13, *p* = .001, partial *η*^*2*^ = .113; time of measurement: *F*(2, 190) = 5.79, *p* = .004, partial *η*^*2*^ = .057). There was no interaction effect (*F*(1.86, 176.37) = .693, *p* = .491). As the Mauchly’s test indicated that the assumption of sphericity had been violated (*χ*^*2*^(2) = 7.56; *p* < .05), degrees of freedom were corrected using Greenhouse Geisser (*ε* = .93). Thus, the estimation of *psychosocial needs* decreased over time and the employees of CAP estimated the children’s *psychosocial needs* higher than those of YWO did (Table 3).

## Discussion

The present study is the first one investigating in a longitudinal survey the evaluation of case specific cooperation and psychosocial needs by employees of both YWO and CAP as well as descriptive data (including psychopathology of children) over a 6-month period cooperation for n = 96 children and their families, receiving any kind of support from both institutions. The possibility to analyze the course of psychopathology of the concerned cases as well as the possible link between ratings of the case responsible employees and the psychopathology is important as case specific accordance or disaccord between both institutions can affect the course of development of children and families receiving support. Furthermore, it can affect the cooperation not only case specifically but also in general.

Within our sample, a high rate of the surveyed parents were separated and nearly two third of the children lived only with a single parent. Moreover, a high number of parents (especially mothers) described own mental disorders (such as depression and agoraphobia). Nearly all of the participating children were treated in a day patient or inpatient clinic. Thus, the present sample consisted of psychosocially high burdened families confirming some of the previously reported risk factors for the development of psychiatric disorders [5, 27].

Accordingly, it is not surprising that the participating children showed high scores of psychopathology within the clinically relevant range and that children and their families received within the 6-month period any kind of support organized by the YWO (e.g. family assistance, support groups, residential living) as well as different types of psychiatric treatment from CAP (e.g. consultation, psychotherapy, day patient or inpatient treatment, medication), depending on the psychiatric disorder, severity, social functioning, etc. The analyses showed that children’s psychopathology, measured with parent- (CBCL) as well as self-ratings (YSR), decreased across the 6-month period. This reduction in psychopathology scores can be due to various factors. It can be assumed that support from CAP as well as YWO had an impact on the improvements of unknown extent. Beyond that, the case specific cooperation or the natural development of children within the 6-month period could have influenced the psychopathology. Comparable studies that also quantitatively investigated groups of cases with wide inclusion criteria (i.e. no restrictions for one or some psychiatric disorders, place of living, type of support and treatment etc.) at the intersection between youth care and mental health services (including CAP) are lacking. Previous studies, especially focusing on residential care, found heterogeneous results for the children’s development, depending on investigated groups and services [45–47].

The present study surveyed case specific communication, total case specific cooperation between the case specific employees of both institutions and psychosocial needs of the child. While there were differences in the evaluations on case specific communication and psychosocial needs between YWO and CAP employees, there was no difference in the evaluation on total case specific cooperation. Furthermore, there were no correlations between the employees’ evaluations and the children’s psychopathology.

On average, at all three times of measurement case responsible employees of CAP estimated psychosocial needs of the child higher than employees of YWO did. This difference can be due to different understandings of and perspectives on psychosocial needs: One may assume that YWO and CAP employees understand different domains under the term ‘psychosocial needs’ (e.g. mental health needs vs. pedagogical needs). They experience different ways, intensities etc. of direct contact with cases, which they evaluated, and may consider divergent samples as a reference (e.g. receiving support from YWO vs. receiving CAP treatment) for their judgement. Analogously, previous reports and expert interviews described similar influencing factors [19–21]. Within the field of residential or foster care some studies exist which examined mental health needs of concerned children and the ability and options of caregivers to identify mental health problems [48–50]. In the study of Mount et al. [49], despite most of caregivers intuitively correctly identified mental health needs of children living in residential or foster care, 23% failed to identify them, raising the risk for long-term problems for the children. Knowledge transfer and exchange between youth welfare services and CAP employees could help to improve the abilities (in both professions) in identifying needs in concerned children.

In German institutions of welfare services, such as YWO, only (time-)extensive measurements exist to evaluate the need for support of children with physical or psychosocial disability (evaluation of various variables such as living modalities, school/work, family/social life, hygiene) (e.g. [51]), in accordance with the International Classification of Functioning [52]. Even though brief and compact instruments bear the risk of information loss and faulty reduction of complex situations, we suggest that a shortened, practicable and specific instrument to rate psychosocial needs would support a common case specific perspective and the progress within the daily routine. For the above-mentioned reasons, within this instrument a standardized and common language for both institutions is necessary.

Concerning cooperation (measured with *case specific communication* and *total case specific cooperation*), YWO and CAP employee ratings remained stable over the 6-month period, respectively. While both institutions rated the cooperation positive, i.e. as ‘(fairly) good’, YWO employees rated the case specific communication better than CAP employees did. Furthermore and against our assumption, the ratings of YWO and CAP employees did not correlate and sometimes, evaluations of case specific communication and cooperative work differed widely or were even of opposing nature. As stated for differences in the evaluation of psychosocial needs, this discrepancy may result from different perspectives and different meanings or reference groups for same terms, too. Salmon and Rapport [21] conducted a thematic analysis and found that within cross-institutional meetings there is a lack of clarification of terminologies. The risk for “talking on cross purposes” can rise. Within our project, CAP employees mentioned critically that the case specific communication process sometimes lacked of both, an exchange regarding task and responsibility distribution and a transparency in decision-making. However, such factors are important for improved cooperation, which was indicated in various previous reports [16, 20, 21, 53]. This is all the more problematic, as in the daily routine with high workloads and time pressure employees have to make quick decisions and have only limited time, i.e. often only for short consultations. Due to the structure and focus of the medical care system (e.g. high workload, financing by health insurances) there is only limited time left for the exchange with other institutions. At the same time, each YWO employee has a high number of assigned cases and experiences time limitations for each responsibility; despite the fact that the main mission and financing of YWO is a systemic, “over-all” and therefore cross-institutional support of children and their families. The negative impact of restricted financial, physical and temporal resources on cooperation in both systems, youth welfare services (including YWO) and CAP, as well as on children’s development has been discussed in several previous studies and reports [15, 19, 25, 27], too.

To overcome the discussed limiting factors, individual projects arose with comparable solutions—e.g. case manager, common structures or trainings (e.g. [22, 23, 54]). In Dresden too, within the present project, a work group was formed, which revised the Agreement of Cooperation between YWO and CAP with focus on clarification of language as well as clearly named and designed workflows for cross-institutional and case specific cooperation (for details see [29]). Furthermore, common trainings to facilitate mutual knowledge and exchange took place between YWO and CAP employees and those of residential care. Unfortunately, due to the aforementioned financial, personal and other limitations it remains difficult to perpetuate these positive efforts as part of a project and transfer them into daily routine.

Another important factor that should be taken into consideration for future cross-institutional cooperation in Germany is that youth welfare services and CAP are subject of separate historical developments, laws and governmental departments. Hence, scope and aim of the institutions and academic backgrounds of the employees are different [55]. In line with that, employees have different perspectives and foci in their work and on the children and their families. A different perspective on the children’s functioning and development may also lead to discrepant evaluations of cooperation. This is typical and enriching, but in cases where both institutions are responsible for the same child’s care, it is important to exchange viewpoints and find a common language and perspective. Furthermore, in case of mentally concerned children it is even more important to consider risk and resilience factors for coordinated treatment and support into remission or even recovery.

Therefore, the necessity remains, to build municipal focus groups or groups of case managers, who develop workflows for cooperation with consideration of common language, organize common meetings or trainings for (knowledge) exchange as well as monitor and supervise the compliance to workflows and availability of necessary resources. Additionally, future research should pursue this topic with quantitative surveys to emphasize and convey the need for further improvement in cooperation into decision-making processes. Furthermore, the evaluation of the success of innovative projects to improve cross-institutional communication and cooperation is a pivotal goal.

## Limitations

Some limitations of the study should be taken into account when interpreting our results. First, the recruiting process as well as the compliance of participants proved to be much more difficult than ever thought (e.g. a lot of families agreed on the telephone to participate but did not appear to study appointments (first as well as consecutive ones) also we had arranged email and short message reminders). We could not systematically assess reasons why families did not agree to participate or missed appointments; reasons that have been named at the telephone were no time for participation, bad experiences etc. Therefore, it can be assumed that the sample was partially selective, so that among other things families with negative experiences or anticipations with both institutions are underrepresented. Nevertheless, this unintended preselection is notable but marginal given the study’s purpose of exploration and identifying case specific cooperation. Secondly, due to compliance issues and missing data we had to use regression imputation, which can lead to type I errors. However, the sample consisted of relevant and high burdened cases receiving support from YWO as well as CAP. Due to the explorative approach, feasibility and ethical considerations, the present study had no control group with children without support by YWO and/or treatment by CAP. Due to this, it could not be explained to which factors (such as time, YWO or CAP support) the described improvements in psychopathology scores can be attributed.

Lastly, due to lack of established instruments and in view of the explorative approach of the present study, we used previous descriptions of process and outcome variables including instruments of cooperation and quality management within the youth welfare and healthcare sector (acc. [37–39]) to develop a questionnaire assessing the quality of cooperation. The different versions were optimized for clarity and comprehensibility by employees of YWO and CAP, but not validated in an extra study. The internal validity of the used score *case specific communication* could be interpreted as good, for the score *total case specific cooperation* as questionable to acceptable.

## Conclusion

The present study investigated cooperation case specifically and for the first time quantitatively, longitudinally, from different perspectives and in relation to children’s psychopathology. In general, cooperation received positive ratings but there was no correlation between the case specific evaluations of YWO and CAP employees. This implies that, on the one hand, there is satisfaction with the cooperation. On the other hand, meaningful differences in case-specific ratings between both institutions exist indicating possibility and need for improvement in regulations and contractual agreements as well as daily work life. It seems important that YWO and CAP know the case specific perspective of each other for a comprehensive and well-coordinated support for the children and their families. Beyond that, cooperation needs the will of the management and more financial investments in staff and time resources—on the one hand to improve quality of treatment and support of the children and their families and on the other hand to protect the (mental) health of case responsible employees. The implementation of more exchange of higher quality and transparency continues to ensure smoother cooperation.

## Data Availability

Questions regarding the datasets of the current study may be addressed to the corresponding author.

## Abbreviations

CAP: child and adolescent psychiatry
YWO: youth welfare office
AoC: agreement of cooperation
CBCL: Child Behavior Checklist
YSR: Youth Self Report

## Footnotes

1 In the following *children* includes also adolescents up to the age of 18 years.

2 In the following *cooperation* stands for cross-institutional cooperation.
